# Case of Hydrocephalus Internus

**Published:** 1806-01

**Authors:** 


### Case of Hydrocephalus Internus; by Dr. Witte.

A Widow Lady, aged 53, mother of several children, and always, as she asserted, in good health, suddenly experienced a disagreeable sensation in her left eye, but which she considered as of no consequence. A few days after she observed a dimness in this eye, which prevented her distinguishing objects even at a short distance. The next morning, after a good night's rest, on rubbing the bulb of the eye with her finger, to promote the power of seeing, she was unable to distinguish the light, every thing appearing quite dark. On examining the eye, the iris was clear, but more dilated than natural, and its contractility entirely lost. In the right eye the contractility was likewise diminished, but in a smaller degree, and the iris a little more dilated. The objects she saw with this eye did not appear quite so clear as before, but but sometimes in a mist, which was soon dispersed when she rubbed the eye with her finger. On enquiring into her previous state of health, she observed, that she had often suffered, for nearly eighteen years past, violent head-ach on one side, which was always increased before her monthly period came on, and that she had consulted a great number of regular physicians as well as quacks, to obtain relief, but without any effect. About five years ago the monthly period ceased, and since that time the head-ach became universal, sometimes stretching towards the shoulder, and accompanied with giddiness. Excepting these complaints she enjoyed a pretty good appetite; her tongue was clean; the body rather bound; the night's rest good; and yet, notwithstanding, she felt a great weariness in all her limbs.

Doctor W. directly formed an opinion, that the source of the affection of the eyes must be in the brain; he therefore informed the relations of the lady, that, probably, the right eye would likewise become blind; that the eyes would be drawn irregularly, and the patient squint; that sickness of the stomach, and vomiting of a bilious matter would follow; and this, probably, owing to a collection of water in the brain. In consequence of this opinion, he ordered the hair to be cut, and a large camphorated blister to be applied over the head, at the same time an inciting glyster to be given; the mercurial ointment to be rubbed into the hands and under the feet; and a decoction of the foxglove prescribed, to be taken inwardly. The next day the right eye became blind, and the turn of the eyes commenced. The medicines were continued. On the fourth day all the symptoms became worse; the head-ach was intolerable, and the patient sometimes delirious; the urine was high coloured and scanty, and she had neither appetite nor thirst; the vomiting began, and occasionally continued until she expired, on the seventh day.

On examining the head, after death, the blood vessels of the membranes of the brain were entirely filled with coagulated dark blood; on the right side, the dura mater was firmly attached to the skull, about the size of a crown-piece. The cortex cerebri, corresponding to this part, was of a greyish black colour, and putrified. The anterior ventricle of the brain was much distended, and contained half a pint of a clear lymphatic fluid; the right ventricle contained about an ounce and a half of fluid; at the base of the brain there was likewise some water found. Excepting these appearances, the brain was quite sound and natural, and likewise all the intestines.

